# Supplementary material for: The Evolution and Characterization of the RNA Interference Pathways in Lophotrochozoa
Source: Genome Biol Evol. 2024 May 7;16(5):evae098. doi: 10.1093/gbe/evae098 (PMC11114477; doi:10.1093/gbe/evae098)
Supplement: evae098_Supplementary_Data [file evae098_supplementary_data.zip › Supplementary_materials.zip!/selected_miRNAs/NoGe_selected_miRNAs/mirdeep_pdf_files/NMRB01000795.1_Notospermus_geniculatus_strain_Ushimado_scaffold795,_whole_genome_shotgun_sequence_20959.pdf]

Provisional ID	:	NMRB01000795.1_Notospermus_geniculatus_strain_Ushimado_scaffold795,_whole_genome_shotgun_sequence_20959
Score total	:	8366.8
Score for star read(s)	:	3.9
Score for read counts	:	8362.3
Score for mfe	:	1.2
Score for randfold	:	0
Score for cons. seed	:	-0.6
Total read count	:	16414
Mature read count	:	16031
Loop read count	:	0
Star read count	:	383

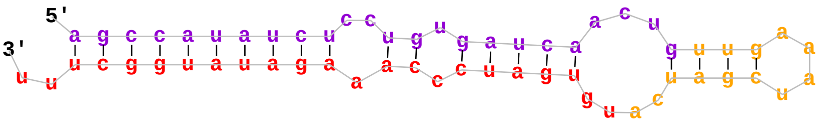

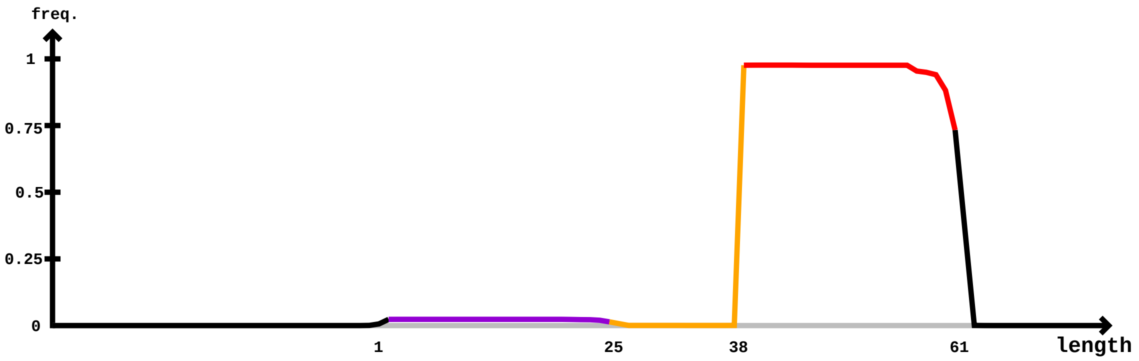


```
                                                  Star                                  Mature
5'- accauaaucccugagagaaagaggugacuccacagccauaucuccugugaucaacuguugaaaucgaucaugugaucccaaagauauggcuuuguauguugccucaag   -3'    obs
    accauaaucccugagagaaagaggugacuccacagccauaucuccugugaucaacuguugaaaucgaucaugugaucccaaagauauggcuuuguauguugccucaag          exp
    ....................((((((((..((.((((((((((..((.(((((...((((....))))....))))).)).))))))))))).))...))))))))...   reads      mm          sample
    ......................................................................ugugaucccaaagauaugg...................   7          0           Fm2
    ......................................................................ugugaucccaaagauauggA..................   1          1           Fm2
    ......................................................................ugugaucccaaagauauggcG.................   1          1           Fm2
    ......................................................................ugugaucccaaagauauggcu.................   6          0           Fm2
    ......................................................................ugugaucccaaagauauggcuu................   2          0           Fm2
    ......................................................................ugugaucccaaagauauggcuuu...............   20         0           Fm2
    ......................................................................ugugaucccaaagauaCggcuuu...............   1          1           Fm2

    ......................................................................ugugaucccaaagauauggcu.................   2          0           Fm1
    ......................................................................ugugaucccaaagauauggcuu................   3          0           Fm1
    ......................................................................ugugaucccaaagauauggcuuu...............   17         0           Fm1

    ...............................acagccauaucuccugugauc........................................................   1          0           Mm2
    ...............................acagccauaucuccugugauca.......................................................   4          0           Mm2
    ...............................acagccauaucuccugugaucaa......................................................   1          0           Mm2
    ...............................acagccauaucuccugugaucaac.....................................................   1          0           Mm2
    ...............................acagccauaucuccugugaucaacu....................................................   2          0           Mm2
    ...............................acagccauaucuccugCgaucaacu....................................................   1          1           Mm2
    ................................cagccauaucuccuguAauc........................................................   1          1           Mm2
    ................................cagccauaucuccugugauc........................................................   6          0           Mm2
    ................................cagccauaucuccugugauca.......................................................   7          0           Mm2
    ................................cagccauaucuccugugaucaa......................................................   2          0           Mm2
    ................................cagccauaucuccugugaucaac.....................................................   12         0           Mm2
    ................................cagccauaucuccugugaucaacu....................................................   30         0           Mm2
    ................................cagccauaucuccugugaucaacG....................................................   1          1           Mm2
    ................................cagccauaucuccugugaucaacug...................................................   5          0           Mm2
    .................................agccauaucuccugugaucaac......................................................   2          0           Mm2
    .................................agccauaucucUugugaucaacu.....................................................   1          1           Mm2
    .................................agccauaucuccugugaucaacu.....................................................   35         0           Mm2
    .................................agccauaucuccugugaGcaacug....................................................   1          1           Mm2
    .................................agccauaucuccugAgaucaacug....................................................   1          1           Mm2
    .................................agccauaucuccugugaCcaacug....................................................   1          1           Mm2
    .................................agccauaucuccugugaAcaacug....................................................   1          1           Mm2
```

```
                                 Star                                Mature
accauaaucccugagagaaagaggugacuccacagccauaucuccugugaucaacuguugaaaucgaucaugugaucccaaagauauggcuuuguauguugccucaag
.................................agccauaucuGcugugaucaacug...................................................	1	1	Mm2
.................................agAcauaucuccugugaucaacug...................................................	1	1	Mm2
.................................agccauaucuccugugaucaacGg...................................................	2	1	Mm2
.................................agccauaucuccugugaucaacug...................................................	158	0	Mm2
.................................agccauaucuccugugaucaacugu..................................................	1	0	Mm2
.................................agccauaucuccugugaucaacuguugaaUu............................................	2	1	Mm2
..................................gccauaucuccugugaucaacug...................................................	1	0	Mm2
.....................................auaucuccugugaucaacug...................................................	1	0	Mm2
.........................................................uugaaaucgaucaugugau................................	4	0	Mm2
.........................................................uugaaaucgaucaugugauc...............................	2	0	Mm2
.....................................................................Cugugaucccaaagauauggcuuu...............	1	1	Mm2
......................................................................ugugaucccaaagauauA....................	1	1	Mm2
......................................................................ugAgaucccaaagauaug....................	1	1	Mm2
......................................................................ugugaucccaaagauauU....................	4	1	Mm2
......................................................................ugugaucccaaagauauC....................	1	1	Mm2
......................................................................ugugaucccaaagauaug....................	168	0	Mm2
......................................................................ugugaucccaaagauaugA...................	1	1	Mm2
......................................................................ugugaucccaaagauaugg...................	27	0	Mm2
......................................................................ugugaucccaaagauaCgg...................	1	1	Mm2
......................................................................ugugaucccaaagauaugU...................	1	1	Mm2
......................................................................ugugauccUaaagauauggc..................	1	1	Mm2
......................................................................ugugaucccaaagauauUgc..................	1	1	Mm2
......................................................................ugugaucccaaagauauggc..................	80	0	Mm2
......................................................................ugugaucccaaagauauggA..................	1	1	Mm2
......................................................................ugugaAcccaaagauauggcu.................	2	1	Mm2
......................................................................ugugaucccaaagauauggcu.................	514	0	Mm2
......................................................................ugugaucccaaagauauCgcu.................	2	1	Mm2
......................................................................ugugaucccaaagauaAggcu.................	1	1	Mm2
......................................................................ugugaucccaGagauauggcu.................	2	1	Mm2
......................................................................ugugaucccaaagauaugCcu.................	1	1	Mm2
......................................................................ugugaucccaaagauCuggcu.................	2	1	Mm2
......................................................................ugugaucccaUagauauggcu.................	1	1	Mm2
......................................................................ugugaucccaaagauaCggcu.................	2	1	Mm2
......................................................................ugugaucccaaagauauggcG.................	7	1	Mm2
......................................................................ugugaucccaaagauauUgcu.................	3	1	Mm2
......................................................................ugugauAccaaagauauggcu.................	1	1	Mm2
......................................................................ugugaucccaaagauaCggcuu................	2	1	Mm2
......................................................................ugugauccUaaagauauggcuu................	2	1	Mm2
......................................................................ugugaCcccaaagauauggcuu................	1	1	Mm2
......................................................................ugugaucccaaagauauAgcuu................	7	1	Mm2
......................................................................ugugaucccaaagauaugUcuu................	2	1	Mm2
......................................................................uAugaucccaaagauauggcuu................	1	1	Mm2
......................................................................ugugaucccaaagauauggUuu................	1	1	Mm2
......................................................................ugugauccAaaagauauggcuu................	1	1	Mm2
......................................................................ugugaucccaaagauCuggcuu................	1	1	Mm2
......................................................................ugugaucccaaaUauauggcuu................	1	1	Mm2
......................................................................ugugaucccaaagauauUgcuu................	7	1	Mm2
......................................................................ugugaucccaGagauauggcuu................	2	1	Mm2
......................................................................uguAaucccaaagauauggcuu................	2	1	Mm2
......................................................................ugugaucAcaaagauauggcuu................	1	1	Mm2
......................................................................ugugaucccaaagauauggcuu................	1583	0	Mm2
......................................................................ugugaucccaaagauaugAcuu................	1	1	Mm2
......................................................................ugugaucccaCagauauggcuu................	3	1	Mm2
......................................................................ugugaucccaaCgauauggcuu................	1	1	Mm2
......................................................................Cgugaucccaaagauauggcuu................	1	1	Mm2
......................................................................ugugaucccaaagauauggcuG................	1	1	Mm2
......................................................................ugugaucccaaagauaGggcuu................	4	1	Mm2
......................................................................uCugaucccaaagauauggcuu................	2	1	Mm2
......................................................................ugugaucccaaagauauCgcuu................	8	1	Mm2
......................................................................ugugaucccaaagauauggcuA................	1	1	Mm2
......................................................................ugugaucccaaagaGauggcuu................	1	1	Mm2
......................................................................ugugaucccaaagauauggAuu................	1	1	Mm2
......................................................................ugugaucccaaagauGuggcuuu...............	2	1	Mm2
......................................................................ugugaucccaaagauauggcCuu...............	5	1	Mm2
......................................................................ugugaucccaCagauauggcuuu...............	11	1	Mm2
......................................................................ugugaucccaaagauauUgcuuu...............	23	1	Mm2
......................................................................ugugaucccaaaAauauggcuuu...............	1	1	Mm2
......................................................................ugugaucccaaagauauggcGuu...............	2	1	Mm2
......................................................................ugugauccUaagauauggcuuu................	1	1	Mm2
......................................................................ugugaucccaaagauaugAcuuu...............	1	1	Mm2
```

```
                                Star                                Mature
accauaaucccugagagaaagaggugacuccacagccauaucuccugugaucaacuguugaaaucgaucaugugaucccaaagauauggcuuuguauguugccucaag
......................................................................ugugauNccaaagauauggcuuu...............   1     1    Mm2
......................................................................ugugaucccaaagauauggcAuu...............   5     1    Mm2
......................................................................ugugaGcccaaagauauggcuuu...............   3     1    Mm2
......................................................................ugugauccUaaagauauggcuuu...............   4     1    Mm2
......................................................................ugugaucUcaaagauauggcuuu...............   7     1    Mm2
......................................................................ugugaucccaaagauCuggcuuu...............   3     1    Mm2
......................................................................ugugaucccaaagauUuggcuuu...............   4     1    Mm2
......................................................................ugugaucccaaagaGauggcuuu...............   7     1    Mm2
......................................................................uguAaucccaaagauauggcuuu...............   3     1    Mm2
......................................................................ugAgaucccaaagauauggcuuu...............   1     1    Mm2
......................................................................ugugaucccCaagauauggcuuu...............   3     1    Mm2
......................................................................ugugaucccaaagauauAgcuuu...............   40    1    Mm2
......................................................................ugugaucccGaagauauggcuuu...............   3     1    Mm2
......................................................................ugugCucccaaagauauggcuuu...............   1     1    Mm2
......................................................................ugugaCcccaaagauauggcuuu...............   1     1    Mm2
......................................................................ugugaucccaaagauaugNcuuu...............   1     1    Mm2
......................................................................ugugaucccaaagauaAggcuuu...............   11    1    Mm2
......................................................................Ngugaucccaaagauauggcuuu...............   2     1    Mm2
......................................................................ugugauUccaaagauauggcuuu...............   3     1    Mm2
......................................................................ugugaucccaaagCuauggcuuu...............   1     1    Mm2
......................................................................ugugaucccaaagauauggcuuC...............   3     1    Mm2
......................................................................ugugaucccaaagauauCgcuuu...............   33    1    Mm2
......................................................................ugugaucccaaagauaGggcuuu...............   14    1    Mm2
......................................................................ugugaucccaaagauaCggcuuu...............   19    1    Mm2
......................................................................ugugaucccaaagauauggcuCu...............   3     1    Mm2
......................................................................uAugaucccaaagauauggcuuu...............   3     1    Mm2
......................................................................ugugaucccaaagauaugCcuuu...............   1     1    Mm2
......................................................................ugugaucccaaagauauggcuGu...............   8     1    Mm2
......................................................................ugGgaucccaaagauauggcuuu...............   8     1    Mm2
......................................................................ugugaNcccaaagauauggcuuu...............   1     1    Mm2
......................................................................ugugaAcccaaagauauggcuuu...............   6     1    Mm2
......................................................................ugugaucccaaGgauauggcuuu...............   6     1    Mm2
......................................................................ugugaucccaaagauauggUuuu...............   3     1    Mm2
......................................................................ugugaucAcaaagauauggcuuu...............   1     1    Mm2
......................................................................ugugaucccaaCgauauggcuuu...............   1     1    Mm2
......................................................................uguUaucccaaagauauggcuuu...............   1     1    Mm2
......................................................................ugugaucccaUagauauggcuuu...............   9     1    Mm2
......................................................................ugugGucccaaagauauggcuuu...............   2     1    Mm2
......................................................................ugugaucccaGagauauggcuuu...............   18    1    Mm2
......................................................................ugugaucccaaaUauauggcuuu...............   1     1    Mm2
......................................................................ugugaucccaaagauauggcuuA...............   6     1    Mm2
......................................................................ugugauGccaaagauauggcuuu...............   1     1    Mm2
......................................................................ugugauccAaaagauauggcuuu...............   2     1    Mm2
......................................................................ugugaucccaaagauauggcuuu...............   8459  0    Mm2
......................................................................Agugaucccaaagauauggcuuu...............   2     1    Mm2
......................................................................ugugaucccaaagauauggcuuG...............   7     1    Mm2
......................................................................ugugaucccaaagauaugUcuuu...............   4     1    Mm2
......................................................................ugCgaucccaaagauauggcuuu...............   4     1    Mm2
......................................................................ugugaucccaaagauauggcuAu...............   3     1    Mm2
......................................................................ugugaucccaaagauauUgcuuug..............   1     1    Mm2
......................................................................ugugaucccaaagauauggcuuug..............   13    0    Mm2
......................................................................ugugaucccaaagauauggcuuuC..............   1     1    Mm2
......................................................................ugugaucccaaagauauCgcuuug..............   1     1    Mm2
......................................................................ugugaucccaaagauauggcuuuA..............   17    1    Mm2
......................................................................ugugaucccaaagauauggcuuuU..............   252   1    Mm2
......................................................................ugugaucccaaagauauggcuuuUu.............   5     1    Mm2
......................................................................ugugaucccaaagauauggcuuuguU............   1     1    Mm2
.......................................................................gugaucccaaagauauggcuuu...............   2     0    Mm2
........................................................................ugaucccaaagauauggcuuu...............   1     0    Mm2

................................cagccauaucuccugugaucaac.....................................................   12    0    Mm1
................................cagccauaucuccugugGucaac.....................................................   1     1    Mm1
................................cagccauaucuccugugaucaacu....................................................   1     0    Mm1
.................................agccauaucuccugugaucaacu....................................................   17    0    Mm1
.................................agccauaucuccugugaucaacug...................................................   7     0    Mm1
......................................................................ugugaucccaaagauauC....................   3     1    Mm1
......................................................................ugugaucccaaagauaug....................   106   0    Mm1
......................................................................ugugaucccaaagauaGg....................   1     1    Mm1
......................................................................ugugaucccaaagauauA....................   3     1    Mm1
......................................................................ugugaucccaaagauaugg...................   31    0    Mm1
```

```
                                Star                                Mature
accauaaucccugagagaaagaggugacuccacagccauaucuccugugaucaacuguugaaaucgaucaugugaucccaaagauauggcuuuguauguugccucaag
......................................................................ugugaucccGaagauaugg...................    1     1     Mm1
......................................................................ugugaucccaaagauauggA..................    8     1     Mm1
......................................................................ugugaucccaaagauauCgc..................    1     1     Mm1
......................................................................ugugaucccaaagauauggc..................    23    0     Mm1
......................................................................uguCaucccaaagauauggcu.................    1     1     Mm1
......................................................................ugugauccUaaagauauggcu.................    1     1     Mm1
......................................................................ugugaucccaaagauauggcu.................    171   0     Mm1
......................................................................ugugaucccaaagauaAggcu.................    1     1     Mm1
......................................................................ugugaucccaaagauCuggcu.................    1     1     Mm1
......................................................................ugugaucccaCagauauggcu.................    1     1     Mm1
......................................................................ugugaucccaaagauauCgcu.................    1     1     Mm1
......................................................................ugugaucccaaagauauAgcuu................    1     1     Mm1
......................................................................ugugUucccaaagauauggcuu................    1     1     Mm1
......................................................................ugugaucccaaagauaCggcuu................    1     1     Mm1
......................................................................Ngugaucccaaagauauggcuu................    1     1     Mm1
......................................................................ugugGucccaaagauauggcuu................    1     1     Mm1
......................................................................ugugaucccaaagCuauggcuu................    1     1     Mm1
......................................................................ugugaucccaaagauauCgcuu................    1     1     Mm1
......................................................................ugugaucccaaagauauggcuu................    255   0     Mm1
......................................................................ugugaucccaaagauauUgcuu................    1     1     Mm1
......................................................................ugugaucccaaagauauggcuuu...............    611   0     Mm1
......................................................................ugugaucccaaagauauCgcuuu...............    5     1     Mm1
......................................................................ugugaucccaUagauauggcuuu...............    1     1     Mm1
......................................................................ugugaucccaGagauauggcuuu...............    2     1     Mm1
......................................................................ugugaucccaaagauaGggcuuu...............    1     1     Mm1
......................................................................ugugaucccaaagauaAggcuuu...............    1     1     Mm1
......................................................................ugugaucccaaagauaCggcuuu...............    2     1     Mm1
......................................................................ugugaucccaaagauauAgcuuu...............    1     1     Mm1
......................................................................ugugaucccaaagauauggcuuuU..............    19    1     Mm1
.......................................................................gugaucccaaagauauggcuuu...............    1     0     Mm1

................................cagccauaucuccugugaucaa......................................................    2     0     Fm3
.................................agccauaucuccugugaucaacug...................................................    1     0     Fm3
......................................................................ugugaucccaaagauaug....................    1     0     Fm3
......................................................................ugugaucccaaagauauggcuu................    3     0     Fm3
......................................................................ugugaucccaaagauauCgcuu................    1     1     Fm3
......................................................................ugugaucccaaagauauggcuuu...............    7     0     Fm3

................................cagccauaucuccugugauc........................................................    1     0     Mm3
................................cagccauaucuccugugaucaac.....................................................    6     0     Mm3
................................cagccauaucuccugugaucaacu....................................................    5     0     Mm3
.................................agccauaucuccugugaucaacu....................................................    4     0     Mm3
.................................Ngccauaucuccugugaucaacug...................................................    1     1     Mm3
.................................agccauaucuccugugaucaacug...................................................    43    0     Mm3
.................................agccauaucuccugugaucaacuU...................................................    1     1     Mm3
.........................................................uugaaaucgaucaugugauc...............................    1     0     Mm3
......................................................................ugugaucccaaGgauaug....................    1     1     Mm3
......................................................................ugugaucccCaagauaug....................    1     1     Mm3
......................................................................ugugaucccaaagauauA....................    1     1     Mm3
......................................................................ugugaucccaaagauaug....................    66    0     Mm3
......................................................................uAugaucccaaagauaug....................    1     1     Mm3
......................................................................ugugaucccaaagauaugg...................    8     0     Mm3
......................................................................ugugaucccaaagauauggA..................    1     1     Mm3
......................................................................ugugaucccUaagauauggc..................    1     1     Mm3
......................................................................ugugaucccaaagauauggc..................    28    0     Mm3
......................................................................ugugaucccaaagauauggcC.................    1     1     Mm3
......................................................................ugugaucccaaagauaGggcu.................    1     1     Mm3
......................................................................ugugaucccaaagauauCgcu.................    1     1     Mm3
......................................................................ugugaucccaaagauauUgcu.................    2     1     Mm3
......................................................................ugugaucccaaagauauggcG.................    1     1     Mm3
......................................................................Agugaucccaaagauauggcu.................    1     1     Mm3
......................................................................ugugaucccaaagauaAggcu.................    1     1     Mm3
......................................................................ugugaucccaaagauauggcu.................    232   0     Mm3
......................................................................ugugaucccaaagauauggcuu................    512   0     Mm3
......................................................................ugugaucccaaagauUuggcuu................    1     1     Mm3
......................................................................uCugaucccaaagauauggcuu................    1     1     Mm3
......................................................................ugugaucccaaagauauggcGu................    1     1     Mm3
......................................................................ugugaucccaUagauauggcuu................    3     1     Mm3
......................................................................ugugaucccaaGgauauggcuu................    2     1     Mm3
......................................................................ugugaucccaaagauauggcuG................    2     1     Mm3
```

Star **Mature**

```
accauaaucccugagagaaagaggugacuccacagccauaucuccugugaucaacuguugaaaucgaucaugugaucccaaagauauggcuuuguauguugccucaag
......................................................................ugugaucccaaagauaAggcuu................ 6 1 Mm3
......................................................................ugugaucccaaagauauCgcuu................ 2 1 Mm3
......................................................................Ngugaucccaaagauauggcuu................ 1 1 Mm3
......................................................................ugugaucccaaagauauUgcuu................ 1 1 Mm3
......................................................................ugugaucccaaagauauAgcuu................ 3 1 Mm3
......................................................................ugugaucccaaagauaGggcuu................ 2 1 Mm3
......................................................................ugugaucccaCagauauggcuuu............... 5 1 Mm3
......................................................................uAugaucccaaagauauggcuuu............... 1 1 Mm3
......................................................................ugugaucAcaaagauauggcuuu............... 1 1 Mm3
......................................................................ugugaucccaaagauaGggcuuu............... 8 1 Mm3
......................................................................uguUaucccaaagauauggcuuu............... 1 1 Mm3
......................................................................ugugaucccUaagauauggcuuu............... 1 1 Mm3
......................................................................ugugaAcccaaagauauggcuuu............... 1 1 Mm3
......................................................................ugugaucccaUagauauggcuuu............... 3 1 Mm3
......................................................................ugugaucccaaagauaCggcuuu............... 5 1 Mm3
......................................................................ugugaucccaGagauauggcuuu............... 3 1 Mm3
......................................................................ugugaCcccaaagauauggcuuu............... 1 1 Mm3
......................................................................ugugaucccaaagGuauggcuuu............... 1 1 Mm3
......................................................................ugugauccUaaagauauggcuuu............... 1 1 Mm3
......................................................................ugugaucccaaCgauauggcuuu............... 1 1 Mm3
......................................................................ugugaucccaaagauauAgcuuu............... 9 1 Mm3
......................................................................uguCaucccaaagauauggcuuu............... 1 1 Mm3
......................................................................uUugaucccaaagauauggcuuu............... 1 1 Mm3
......................................................................ugugaucccaaagauauggcuuu............... 2121 0 Mm3
......................................................................ugugaucccaaagauaAggcuuu............... 4 1 Mm3
......................................................................ugugGucccaaagauauggcuuu............... 1 1 Mm3
......................................................................ugugaucccaaagauauggcuuG............... 3 1 Mm3
......................................................................ugugaucccaaagauauCgcuuu............... 13 1 Mm3
......................................................................ugugaucccaaagaAauggcuuu............... 1 1 Mm3
......................................................................ugugaucccaaagauauggUuuu............... 1 1 Mm3
......................................................................ugugaucccaaUgauauggcuuu............... 2 1 Mm3
......................................................................ugugaucccaaagauauUgcuuu............... 3 1 Mm3
......................................................................ugugaucccaaagauauggcuuA............... 1 1 Mm3
......................................................................ugugaucccaaagauaugAcuuu............... 2 1 Mm3
......................................................................ugugaucGcaaagauauggcuuu............... 1 1 Mm3
......................................................................ugugaucccaaagauaugUcuuu............... 1 1 Mm3
......................................................................ugugaucccaaagaGauggcuuu............... 2 1 Mm3
......................................................................ugugaucccaaagauauggcuuuA.............. 2 1 Mm3
......................................................................ugugaucccaaagauauggcuuuU.............. 67 1 Mm3
......................................................................ugugaucccaaagauauggcuuug.............. 2 0 Mm3
......................................................................ugugaucccaaagauauggcuuuUu............. 2 1 Mm3
.......................................................................gugaucccaaagauauggcuuu............... 2 0 Mm3
```
